# Out of sight, out of mind, but at high risk: incidence, timing and risk factors for hyperkalemia after liver transplant discharge

**DOI:** 10.3389/fimmu.2026.1924687

**Published:** 2026-10-07

**Authors:** Peyton Crest, Holland Stacey, Akshara Vykunta, Ella Atsavapranee, Kevin Kachi Ogbonna, Kara Ushijima, Liliana Espinosa, Nathan Robbins, Dieter Adelmann, Garrett R. Roll

**Affiliations:** 1 Division of Transplant, Department of Surgery, University of California, San Francisco, San Francisco, CA, United States; 2 University of California, San Francisco School of Medicine, San Francisco, CA, United States; 3 Department of Anesthesia and Perioperative Care, University of California, San Francisco, San Francisco, CA, United States

**Keywords:** calcineurin inhibitor, hyperkalemia, liver transplantation, post-transplant complications, renal dysfunction

## Abstract

**Background:**

Hyperkalemia occurs in liver transplant (LT) recipients due to calcineurin inhibitor initiation and post-operative renal dysfunction and is associated with significant patient morbidity and resource expenditures. Little is known about the prevalence and timing of hyperkalemia after LT discharge.

**Methods:**

Adults undergoing LT between 1/2022-12/2024 at a single transplant center were included, excluding simultaneous heart-liver transplant recipients and patients who died before discharge. The primary exposure was time to hyperkalemia (serum potassium > 5.0 mmol/L) within three months of LT discharge. Univariable and multivariable Cox proportional hazards regression was used to identify predictors of hyperkalemia.

**Results:**

Of 544 patients, 268 (49%) developed hyperkalemia within three months following LT discharge. Compared to patients without hyperkalemia, those who developed hyperkalemia were more likely to have diabetes mellitus (30% vs 20%, p=0.01) and higher laboratory MELD scores (29 vs 24, p=0.02). The median time to the first hyperkalemia episode was 11 days (IQR 5-28) after LT discharge, with the highest incidence occurring during the first week after discharge. 110 patients (20%) had hyperkalemia documented during readmission. On multivariable Cox regression, CKD stages 3a (aHR 1.63, 95% CI 1.11-2.40), 3b (aHR 2.38, 95% CI 1.51-3.74), and 5 (aHR 2.01, 95% CI 1.32–3.06); one week pre-transplant potassium (aHR 1.52, 95% CI 1.24-1.84); and discharge potassium (aHR 1.63, 95% CI 1.24-2.16) were independently associated with hyperkalemia.

**Conclusion:**

Hyperkalemia is common within the first three months following LT discharge. These findings support the development of risk-stratified surveillance and management guidelines to reduce hyperkalemia-related morbidity and healthcare utilization. Given the cost and access barriers associated with outpatient hyperkalemia treatment, targeted interventions are needed to safeguard patients after discharge.

## 1 Introduction

End-stage liver disease (ESLD) is characterized by significant metabolic dysfunction, including electrolyte abnormalities. Following liver transplantation (LT), these disturbances may persist or evolve in the setting of calcineurin inhibitor (CNI) initiation and fluctuating renal function (1). Hyperkalemia, in particular, is common after LT and associated with increased healthcare utilization, patient morbidity, and even mortality (2).

Hyperkalemia is a well-described complication during the intraoperative period, particularly following reperfusion, where potassium loads from preservation solution and blood transfusion are recognized contributors (3). In contrast, post-transplant hyperkalemia is driven by acute or chronic kidney dysfunction and medication effects. Although hyperkalemia has been reported up to 12 months after LT, data describing the frequency of post-transplant hyperkalemia are limited and inconsistent (4–6). For example, a retrospective study from the early tacrolimus era reported that 54% of LT recipients required treatment for hyperkalemia within 12 months of transplantation, whereas a separate cohort observed hyperkalemia in 25% of patients without pre-existing renal dysfunction over the same interval (5, 6). These differences likely reflect heterogeneity in study era, immunosuppression regimens, patient populations, and outpatient follow-up practices.

Several factors may contribute to post-transplant hyperkalemia, including CNI exposure, impaired renal function (including chronic kidney disease [CKD] or acute kidney injury [AKI]), and concomitant medications (7). However, the relative contribution of these factors and the timing of hyperkalemia following discharge remain incompletely characterized. Earlier identification of patients at risk for hyperkalemia may facilitate targeted interventions, including increased outpatient monitoring, dietary modifications, immunosuppression management, and prophylactic use of potassium-lowering medications. These interventions, in turn, have the potential to reduce hyperkalemia-related morbidity and healthcare utilization.

Given these knowledge gaps, we evaluated the frequency of hyperkalemia following discharge after LT. The primary objective of this study was to assess the incidence and timing of hyperkalemia within three months of LT discharge. Secondary objectives identified predictors of hyperkalemia after LT discharge and evaluated the frequency of hospital readmissions with hyperkalemia, including readmissions in which hyperkalemia was a primary or secondary diagnosis.

## 2 Methods

### 2.1 Study design and population

This single-center, retrospective cohort study included all adult patients who underwent LT at a quaternary academic transplant center between January 1, 2022 and December 31, 2024. The study end date was selected to ensure a minimum of three months of follow-up for all included patients. Recipients of simultaneous liver-kidney transplants were included, but heart-liver transplant recipients were excluded. No liver-lung or liver-intestine transplants were performed during the study period. Patients who died prior to LT discharge were also excluded. **Supplementary Figure S1** in the **Supplementary Material** provides a detailed population workflow.

Baseline demographic, clinical, and laboratory data were collected through electronic medical record review. Additional data included transplant and donor characteristics, prescribed medications, discharge diet, and hospital readmissions.

This study was approved by the University of California, San Francisco Institutional Review Board and received a waiver of informed consent (IRB #20-31396). All research was conducted in accordance with the Declarations of Helsinki and Istanbul.

### 2.2 Institutional post-transplant care

All patients received standardized institutional post-transplant care. Recipients were maintained on triple-drug immunosuppression with tacrolimus, mycophenolate mofetil, and prednisone. Prednisone was weaned to 5 mg over 6 weeks, and tacrolimus trough levels were maintained between 8–10 ng/mL, with lower levels (6–8 ng/mL) used in patients with renal dysfunction. Outpatient laboratory monitoring followed a standardized schedule: twice weekly for the first 4–8 weeks after discharge, followed by weekly testing for an additional 6–12 weeks. Antifungal prophylaxis included fluconazole 100 mg weekly unless the patient had an indication for another dose due to elevated risk of coccidioidomycosis or the presence of an active fungal infection. All patients received trimethoprim-sulfamethoxazole, dosed as double-strength three times weekly for three months after transplantation. Initiation of medications to reduce serum potassium was at the discretion of the medical team, and no hyperkalemia prevention protocol existed.

### 2.3 Primary outcome definition

Hyperkalemia was defined as serum potassium concentration above the upper limit of the normal reference range at our institution (> 5.0 mmol/L). Episodes occurring during transplant or the remainder of the index hospitalization were not included in this analysis. The primary exposure of interest was the time to first hyperkalemia within three months of LT discharge. For patients with multiple measurements, the earliest value after discharge exceeding the 5.0 mmol/L threshold was used to define hyperkalemia. The peak serum potassium level within the first three months after discharge was also captured.

Hospital readmissions for hyperkalemia were defined as those in which hyperkalemia was listed as the primary diagnosis. Hyperkalemia occurring during readmissions in which it was not the primary diagnosis was also recorded.

### 2.4 Variable definitions

Early allograft dysfunction was defined according to Olthoff et al. (8) CKD stage was assessed using the Kidney Disease: Improving Global Outcomes (KDIGO) criteria and based on estimated glomerular filtration rate (eGFR) using the 2021 CKD-EPI equation (9). Post-operative AKI stage was classified by KDIGO AKI diagnostic criteria using changes in serum creatinine (10).

Dialysis exposure was categorized into three time points. Dialysis prior to transplant was defined as initiation of dialysis or ongoing maintenance dialysis at the time of admission for transplantation. Required inpatient dialysis after transplant was defined as any dialysis session initiated after transplantation and prior to LT discharge. Dialysis after discharge was defined as maintenance dialysis initiated within three months after discharge.

Medication exposures were also captured at three time points. Medications prior to transplant were defined as active medications within one month prior to LT. Medications at discharge were those recorded on the discharge medication reconciliation. Medications after discharge were defined as medications newly prescribed after discharge through three months of follow-up or discharge medications that were continued during this interval. Among patients with hyperkalemia, only medications initiated prior to the onset of hyperkalemia were included.

One-month pre-transplant laboratory values were those closest to 30 days pre-transplant (± 7 days), and one week pre-transplant laboratory values were those closest to 7 days pre-transplant (± 7 days).

### 2.5 Statistical analysis

Categorical variables were summarized as counts and frequencies (%) and compared using Pearson’s chi-squared test. Continuous variables were summarized as medians with interquartile ranges (IQR) and compared using the Wilcoxon rank-sum test.

The primary study outcome was the time from LT discharge to the development of hyperkalemia or last follow-up. Patients without hyperkalemia were censored at the earliest of the following: last known follow-up date, death, graft failure, or manually censored at three months post-transplant. Univariable Cox proportional hazards regression models were used to identify predictors of post-transplant hyperkalemia. Medications after discharge were modeled as time-varying covariates. Variables with p < 0.10 on univariable analysis were considered for inclusion in the multivariable Cox proportional hazards model, which was constructed using a backward stepwise selection approach. The proportional hazards assumption was assessed for all variables in the final multivariable Cox model using scaled Schoenfeld residuals, with visual inspection of residual plots used to evaluate any variables flagged by the global Schoenfeld test. To minimize collinearity, when two or more candidate variables were closely related, a single representative variable was retained based on clinical judgement. Two sensitivity analyses were performed to assess the robustness of findings: one restricted to isolated liver transplant recipients and one applying an alternative hyperkalemia threshold of > 5.5 mmol/L (11).

A p-value of less than 0.05 was considered statistically significant. Analyses were performed using Stata, version 19.0 (StataCorp, College Station, TX).

## 3 Results

### 3.1 Baseline demographics

Baseline characteristics of the study population, stratified by hyperkalemia status, are shown in **Table 1**. Among 544 included patients, the median follow-up time was 717 days (IQR 465-1023), and most (97%) patients had at least 90 days of follow-up. Nearly half (49%) developed hyperkalemia within three months of LT discharge. Patients with hyperkalemia were mostly male (65%), with a median age of 60 years and a median laboratory MELD of 29 at transplant. Predominant etiologies in this group included alcohol-associated liver disease (43%) followed by metabolic dysfunction-associated steatotic liver disease/metabolic dysfunction-associated steatohepatitis (24%).

**Table 1:** Baseline demographics and transplant clinical characteristics, stratified by hyperkalemia status.

Variable	Hyperkalemia n=268	No hyperkalemia n=276	p-value
Patient characteristics			
Age at transplant (years)	60 (51-65)	57 (48-65)	0.05
BMI	27.8 (24.0-32.2)	28.0 (24.3-32.9)	0.54
Sex			0.03
Female	94 (35%)	122 (44%)	
Male	174 (65%)	154 (56%)	
Race/Ethnicity			0.03
White	93 (35%)	126 (46%)	
Black	12 (4%)	13 (5%)	
Hispanic/Latino	110 (41%)	98 (36%)	
Asian	44 (16%)	34 (12%)	
American Indian/ Alaska Native	7 (3%)	1 (0.4%)	
Native Hawaiian/ Other Pacific Islander	2 (0.8%)	4 (1%)	
Comorbidities			
Diabetes mellitus	80 (30%)	54 (20%)	0.01
Hypertension	68 (25%)	62 (22%)	0.43
Hyperlipidemia	38 (14%)	30 (11%)	0.24
Etiology			0.04
Alcohol-associated	114 (43%)	104 (38%)	
HBV	13 (5%)	23 (8%)	
HCV	33 (12%)	24 (9%)	
Cholestasis	14 (5%)	30 (11%)	
Autoimmune	2 (0.8%)	7 (3%)	
MASLD/MASH	64 (24%)	58 (21%)	
Malignancy	3 (1%)	7 (3%)	
Other	25 (9%)	23 (8%)	
Transplant details			
Laboratory MELD at transplant	29 (16-35)	24 (14-33)	0.02
SLK	34 (13%)	20 (7%)	0.03
Donor age (years)	41 (31-50)	40 (30-52)	0.93
NMP	73 (27%)	80 (29%)	0.65
CIT (hours)	8.1 (6.3-11.5)	7.7 (5.3-11.5)	0.14
EBL (L)	2.5 (1.5-5.0)	2.0 (1.0-4.5)	0.02
Donor Type			0.20
DBD	196 (73%)	185 (67%)	
DCD	40 (15%)	44 (16%)	
Living	32 (12%)	47 (17%)	

BMI, Body mass index; HBV, Hepatitis B virus; HCV, Hepatitis C virus; MASLD/MASH, Metabolic dysfunction-associated steatotic liver disease/Metabolic dysfunction-associated steatohepatitis; MELD, Model for End-Stage Liver Disease; SLK, Simultaneous liver-kidney transplant; DBD, Donation after brain death; DCD, Donation after circulatory death; NMP, Normothermic machine perfusion; CIT, Cold ischemia time; EBL, Estimated blood loss.

Compared to patients without hyperkalemia, those who developed hyperkalemia were more likely to have diabetes mellitus (30% vs 20%, p=0.01), higher laboratory MELD scores (29 vs 24, p=0.02), and greater estimated blood loss at transplant (2.5 vs 2.0 L, p=0.02). Additionally, patients with hyperkalemia were more likely to undergo simultaneous liver-kidney transplantation (13% vs 7%, p=0.03).

A sensitivity analysis comparing patients with hyperkalemia defined as serum potassium > 5.5 mmol/L is shown in **Supplementary Table S1** and produced similar results, with the exception of SLK, which was no longer significant.

### 3.2 Perioperative course, renal function, and medications

Perioperative characteristics, laboratory values, and medication exposures are presented in **Table 2**. Notably, patients with post-transplant hyperkalemia had significantly worse renal function across all time points compared to those without, including higher rates of dialysis and lower eGFR values. In the immediate post-operative period, AKI was more common among patients with hyperkalemia, driven primarily by stage 3 AKI (49% vs 31% of overall cohorts, p<0.001). Early allograft dysfunction was also more frequent in this group (23% vs 15%, p=0.03).

**Table 2:** Perioperative characteristics, medications, and laboratory values, stratified by hyperkalemia status.

Variable	Hyperkalemia n=268	No hyperkalemia n=276	p-value
Dialysis			
Dialysis prior to transplant	72 (27%)	43 (16%)	0.001
Required inpatient dialysis after transplant	104 (39%)	62 (22%)	<0.001
Dialysis after discharge	36 (13%)	18 (7%)	0.007
Post-operative AKI*			
Any	232 (87%)	200 (72%)	<0.001
Stage 1†	67 (29%)	71 (36%)	
Stage 2†	35 (15%)	44 (22%)	
Stage 3†	130 (56%)	85 (43%)	
Post-operative outcomes			
EAD	61 (23%)	42 (15%)	0.03
LOS	12 (8-21)	10 (7-17)	<0.001
Discharge diet			0.12
Regular	158 (59%)	183 (66%)	
Renal	51 (19%)	33 (12%)	
Diabetic	37 (14%)	40 (14%)	
Other	22 (8%)	20 (7%)	
Medications prior to transplant			
Bicarbonate	47 (18%)	27 (10%)	0.008
Patiromer calcium sorbitex	2 (1%)	0 (0%)	0.15
Sodium polystyrene sulfonate	2 (1%)	0 (0%)	0.15
Sodium zirconium cyclosilicate	28 (10%)	18 (7%)	0.10
Furosemide During Transplant	188 (70%)	182 (66%)	0.29
Medications prior to discharge			
Bicarbonate	60 (22%)	32 (12%)	0.001
Patiromer calcium sorbitex	0 (0%)	1 (0.4%)	0.32
Sodium polystyrene sulfonate	3 (1%)	1 (0.4%)	0.30
Sodium zirconium cyclosilicate	75 (28%)	53 (19%)	0.02
Medications at discharge			
ACE inhibitor	2 (0.8%)	1 (0.4%)	0.55
Beta blocker	54 (20%)	40 (14%)	0.08
Furosemide	47 (18%)	54 (20%)	0.54
Insulin	94 (35%)	81 (29%)	0.15
Spironolactone	1 (0.4%)	1 (0.4%)	0.98
Trimethoprim- sulfamethoxazole	239 (89%)	247 (89%)	0.91
Medications after discharge			
Bicarbonate	48 (18%)	33 (12%)	0.05
Sodium polystyrene sulfonate	4 (1%)	2 (1%)	0.39
Sodium zirconium cyclosilicate	23 (9%)	10 (4%)	0.02
One month pre-transplant labs			
Creatinine‡	1.1 (0.8-1.4)	0.9 (0.7-1.1)	<0.001
eGFR‡	69 (47-96)	86 (60-111)	<0.001
Potassium	4.3 (3.8-4.7)	4.0 (3.8-4.4)	0.01
One week pre-transplant labs			
Creatinine†	1.1 (0.8-1.6)	0.9 (0.7-1.2)	<0.001
eGFR†	76 (47-98)	87 (61-105)	<0.001
Potassium	4.1 (3.8-4.5)	3.9 (3.6-4.2)	<0.001
Tacrolimus Trough POD5	5.9 (4.2-8.0)	6.3 (4.8-8.0)	0.08
Labs at discharge			
Tacrolimus trough	7.5 (5.9-9.2)	7.7 (6.0-9.3)	0.59
eGFR§	78 (55-99)	93 (66-105)	<0.001
AST	27 (18-42)	32 (20-53)	0.002
ALT	70 (32-129)	95 (51-179)	<0.001
Potassium	4.2 (3.9-4.6)	4.1 (3.8-4.4)	0.001
CKD stage at discharge∥			<0.001
Stage 1	69 (26%)	124 (45%)	
Stage 2	65 (24%)	71 (26%)	
Stage 3a	50 (19%)	34 (12%)	
Stage 3b	30 (11%)	16 (6%)	
Stage 4	14 (5%)	12 (4%)	
Stage 5	40 (15%)	19 (7%)	

^*^Patients with pre-transplant dialysis were considered AKI stage 3.

^†^Of patients with post-operative AKI.

^‡^Patients on dialysis prior to transplant were excluded.

^§^Patients on inpatient dialysis after transplant were excluded.

^∥^Patients on dialysis at or following discharge were considered CKD stage 5.

Among patients with hyperkalemia, medications after discharge are those initiated prior to hyperkalemia onset.

EAD, Early allograft dysfunction; LOS, length of stay; ACE, Angiotensin-converting enzyme; eGFR, estimated Glomerular Filtration Rate; POD, Post-operative day; AST, Aspartate aminotransferase; ALT, Alanine aminotransferase; CKD, Chronic kidney disease.

Serum potassium levels were modestly but significantly higher among patients with hyperkalemia at all pre- and post-transplant time points. While discharge medications were broadly similar between groups, patients with hyperkalemia were more frequently administered bicarbonate and sodium zirconium cyclosilicate prior to discharge and sodium zirconium cyclosilicate after discharge, consistent with management of elevated potassium in this group.

Most patients with hyperkalemia (98%) and without hyperkalemia (99%) were prescribed 100 mg fluconazole weekly, which was continued following discharge. Of those with weekly doses greater than 100 mg (n=9), five (56%) developed hyperkalemia. Of these five patients, most had preserved or mildly reduced renal function at discharge (one with CKD stage 1, three with CKD stage 2, and one with CKD stage 3a) and a median serum potassium of 4.3 mmol/L (IQR 3.9-4.5) at discharge.

A sensitivity analysis comparing patients with hyperkalemia defined as serum potassium > 5.5 mmol/L is shown in **Supplementary Table S2**. Results were similar, with the exception of EAD, which was no longer significant.

### 3.3 Timing of hyperkalemia and readmission course

The median time to the first episode of hyperkalemia was 11 days (IQR 5-28) after LT discharge, with the highest incidence occurring during the first week after discharge, when 38% of cases were observed (**Figure 1**). Among those with hyperkalemia, the median first recorded serum potassium elevation was 5.3 mmol/L (IQR 5.2-5.5), and the median peak serum potassium was 5.6 mmol/L (IQR 5.4-5.9). Across peak serum potassium measurements, 221 patients (82%) had mild elevations (5.1-6.0 mmol/L), 39 (15%) had moderate elevations (6.1-7.0 mmol/L), and 8 patients (3%) had severe elevations (> 7.0 mmol/L).

**Figure 1:**
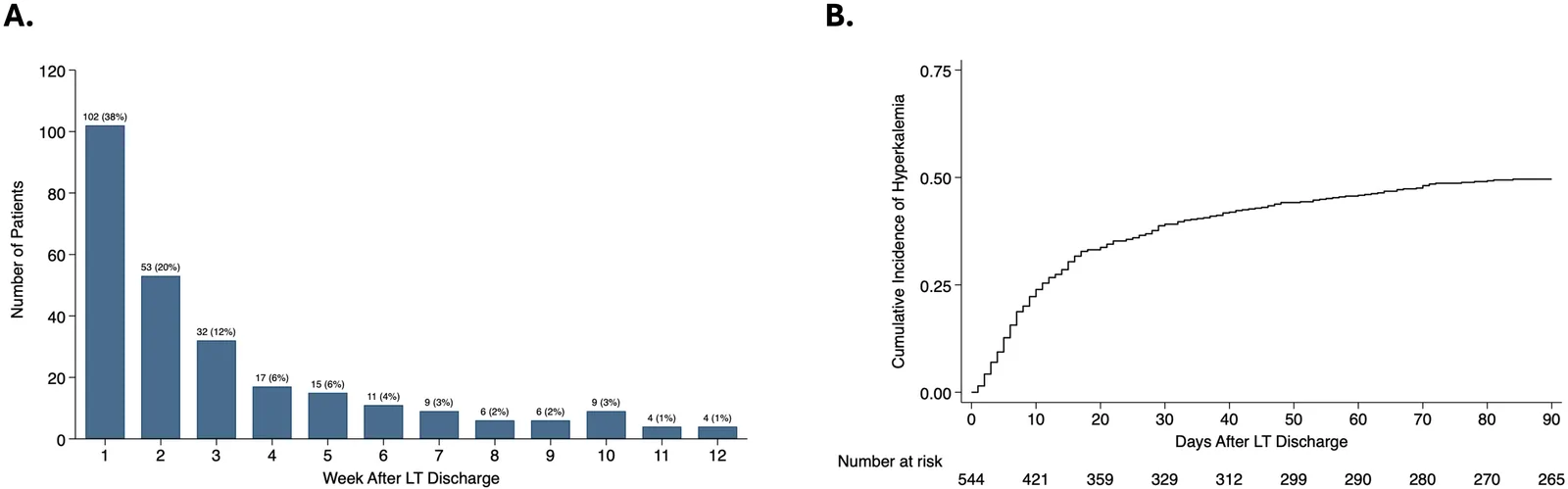
Timing of first hyperkalemia event following liver transplant (LT) discharge based on **(A)** week after discharge and **(B)** cumulative incidence.

Twenty-three patients (9% of those with hyperkalemia; 4% of the overall cohort) presented to the emergency department or were readmitted with hyperkalemia as the primary diagnosis. However, this likely underestimates the true burden of disease, as outside-system encounters could not be fully captured. Among all patients readmitted within three months, 110 (25% of patients with readmission; 20% of the overall cohort) had hyperkalemia documented during the readmission, with a median serum potassium of 5.6 mmol/L (IQR 5.3–5.9).

### 3.4 Predictors of post-transplant hyperkalemia

On univariable Cox regression (**Table 3**), demographic factors significantly associated with hyperkalemia after discharge included recipient age and male sex; however, only sex remained significant on multivariable analysis. Laboratory MELD at transplant and EAD were also significant predictors on univariable analysis but were not significant in the multivariable model.

**Table 3:** Univariable and multivariable Cox regression for time to first hyperkalemia event within three months of post-transplant discharge.

Variable	Univariable HR (95% CI)	p-value	Multivariable aHR (95% CI)	p-value
Patient characteristics				
Age at transplant (per year increase)	1.01 (1.00-1.02)	0.01	1.01 (1.00-1.02)	0.18
Sex, female reference	1.34 (1.04-1.72)	0.02	1.36 (1.05-1.76)	0.02
BMI	0.99 (0.97-1.01)	0.48		
Diabetes mellitus	1.46 (1.12-1.89)	0.005	1.42 (1.09-1.87)	0.01
Hypertension	1.16 (0.88-1.53)	0.28		
Hyperlipidemia	1.25 (0.89-1.77)	0.20		
Ethnicity, white reference				
Black	1.14 (0.62-2.07)	0.68		
Hispanic/Latino	1.34 (1.02-1.77)	0.04		
Asian	1.44 (1.01-2.06)	0.05		
American Indian/ Alaska Native	2.74 (1.27-5.91)	0.01		
Native Hawaiian/ Other Pacific Islander	0.68 (0.17-2.78)	0.60		
Liver disease characteristics				
Laboratory MELD at transplant (per point increase)	1.01 (1.00-1.02)	0.02	1.01 (0.99-1.02)	0.28
Potassium One Week Pre-Tx	1.66 (1.39-1.97)	<0.001	1.52 (1.24-1.84)	<0.001
Etiology, autoimmune reference				
Alcohol-associated	2.97 (0.73-12.04)	0.13		
HBV	1.77 (0.40-7.85)	0.45		
HCV	3.31 (0.79-13.80)	0.10		
Cholestasis	1.48 (0.34-6.53)	0.60		
MASLD/MASH	2.88 (0.71-11.78)	0.14		
Malignancy	1.46 (0.24-8.76)	0.68		
Other	2.94 (0.70-12.40)	0.14		
Transplant characteristics				
SLK	1.59 (1.11-2.28)	0.01		
Donor type, DBD reference				
DCD	0.93 (0.66-1.30)	0.66		
Living	0.73 (0.50-1.06)	0.10		
Donor age (per year increase)	1.00 (0.99-1.01)	0.92		
Transplant Details				
CIT (per hour increase)	1.01 (0.99-1.03)	0.35		
EBL (per liter increase)	1.02 (1.00-1.05)	0.10		
EAD	1.37 (1.03-1.83)	0.03	1.14 (0.84-1.54)	0.40
Renal function				
Creatinine* One Week Pre-Tx (per one unit increase)	1.24 (1.08-1.44)	0.003		
Dialysis prior to transplant	1.56 (1.19-2.04)	0.001		
Required inpatient dialysis after transplant	1.69 (1.32-2.16)	<0.001		
Dialysis after discharge	1.46 (1.03-2.08)	0.03		
eGFR§ at discharge (per one unit increase)	0.99 (0.98-0.99)	<0.001		
Post-operative AKI, no AKI reference†				
Stage 1	1.90 (1.23-2.92)	0.004		
Stage 2	1.77 (1.09-2.89)	0.02		
Stage 3	2.65 (1.78-3.94)	<0.001		
CKD stage at discharge, stage 1 reference‡				
Stage 2	1.48 (1.05-2.07)	0.02	1.13 (0.79-1.63)	0.51
Stage 3a	2.02 (1.40-2.91)	<0.001	1.63 (1.11-2.40)	0.01
Stage 3b	2.56 (1.67-3.93)	<0.001	2.38 (1.51-3.74)	<0.001
Stage 4	1.92 (1.08-3.42)	0.03	1.59 (0.88-2.87)	0.13
Stage 5	2.23 (1.51-3.30)	<0.001	2.01 (1.32-3.06)	0.001
Laboratory values at discharge				
AST (per one unit increase)	0.99 (0.99-1.00)	0.01		
ALT (per one unit increase)	1.00 (1.00-1.00)	0.01		
Tacrolimus trough (per one unit increase)	0.99 (0.94-1.04)	0.60		
Potassium (per one unit increase)	1.78 (1.37-2.32)	<0.001	1.63 (1.24-2.16)	0.001
Diet at discharge & medications				
Renal diet vs other diets	1.49 (1.08-2.05)	0.02		
Sodium zirconium cyclosilicate prior to discharge	1.47 (1.13-1.92)	0.005		
Bicarbonate prior to discharge	1.73 (1.30-2.31)	<0.001		
Beta blockers at discharge	1.34 (0.99-1.80)	0.06		
Furosemide at discharge	0.84 (0.61-1.15)	0.28		
Insulin at discharge	1.21 (0.94-1.55)	0.14		
Medications after discharge∥				
Bicarbonate after discharge	2.04 (1.49-2.79)	<0.001		
Sodium polystyrene sulfonate after discharge	2.57 (0.95-6.89)	0.06		
Sodium zirconium cyclosilicate after discharge	2.84 (1.85-4.35)	<0.001		

^*^Patients on dialysis prior to transplant were excluded.

^†^Patients with pre-transplant dialysis were considered AKI stage 3.

^‡^Patients on dialysis at or following discharge were considered CKD stage 5.

^§^Patients on inpatient dialysis after transplant were excluded.

^∥^Medications after discharge were modeled as time-varying covariates.

Tx, Transplant; HBV, Hepatitis B virus; HCV, Hepatitis C virus; MASLD/MASH, Metabolic dysfunction-associated steatotic liver disease/Metabolic dysfunction-associated steatohepatitis; MELD, Model for End-Stage Liver Disease; SLK, Simultaneous liver-kidney transplant; DBD, Donation after brain death; DCD, Donation after circulatory death; CIT, Cold ischemia time; EBL, Estimated blood loss; EAD, Early allograft dysfunction; CKD, Chronic Kidney Disease; eGFR, estimated Glomerular Filtration Rate; AST, Aspartate aminotransferase; ALT, Alanine aminotransferase.

Renal function was a strong predictor of hyperkalemia, with both pre- and post-transplant markers associated with increased risk. Higher CKD stages at discharge were generally associated with post-transplant hyperkalemia on multivariable analysis; however, both stage 2 and stage 4 were not associated with significant risk. Notably, tacrolimus trough at discharge was not associated with hyperkalemia, nor was tacrolimus trough on post-operative day five (HR 0.97, 95% CI 0.93-1.02, p=0.24).

Serum potassium was a consistent predictor at both pre- and post-transplant time points. On univariable analysis, higher serum potassium at both one-month (HR 1.78, 95% CI 1.29-2.45, p<0.001) and one week pre-transplant (HR 1.66, 95% CI 1.39-1.97, p<0.001) was significantly associated with post-transplant hyperkalemia, as was serum potassium at discharge (HR 1.78, 95% CI 1.37-2.32, p<0.001). Pre-transplant and discharge potassium were weakly correlated (r=0.16) and retained in the multivariable model, in which both remained independently associated with hyperkalemia.

Among after discharge therapies prescribed prior to hyperkalemia onset, patients who required bicarbonate (HR 2.04, 95% CI 1.49-2.79, p<0.001) and sodium zirconium cyclosilicate (HR 2.84, 95% CI 1.85-4.35, p<0.001) were at higher risk for hyperkalemia on univariable analysis, but neither was included in the multivariable model due to confounding by indication, as most were initiated in response to hyperkalemia or borderline serum potassium elevations.

A sensitivity analysis for predictors of the time to hyperkalemia, with hyperkalemia defined as serum potassium > 5.5 mmol/L, is shown in **Supplementary Table S3**. While SLK was not included in the final multivariable model to minimize collinearity, it was significantly associated with hyperkalemia on univariable analysis (HR 1.62, 95% CI 1.01-2.60, p=0.04). Age and sex were not significant on univariable analysis. Notably, in the multivariable model, after adjusting for diabetes, laboratory MELD, serum potassium at one week pre-transplant, and estimated blood loss, both serum potassium at discharge and CKD stages 2–4 were not associated with hyperkalemia (> 5.5 mmol/L).

### 3.5 Isolated liver transplant recipients

A sensitivity analysis restricted to isolated liver transplant recipients (n=490) produced results similar to the total cohort (**Supplementary Tables S4**–**S6**). Sex; diabetes mellitus; serum potassium at one week pre-transplant and at discharge; and CKD stages 3a, 3b, and 5 were independently associated with the time to post-transplant hyperkalemia (serum potassium > 5.0 mmol/L).

## 4 Discussion

Hyperkalemia is associated with significant healthcare utilization and is well characterized in the intraoperative phase of care during LT (4, 12). However, little is known about the incidence of hyperkalemia after LT discharge, which manifests through distinct mechanisms. To date, no studies have described the time to the first episode of hyperkalemia in LT recipients. We sought to address this gap through a contemporary cohort analysis, examining its incidence, severity, and risk factors.

In our cohort of 544 patients, nearly half developed serum potassium elevations > 5.0 mmol/L within three months of discharge, with most cases occurring within the first two weeks following discharge. These results highlight the substantial burden of hyperkalemia in the early post-transplant period, and the clustering of early events suggests that patients are particularly susceptible to electrolyte disturbances during this narrow window following LT for multiple reasons.

Not surprisingly, impaired renal function was a significant predictor of hyperkalemia, among both isolated liver transplant recipients and the total cohort. Markers both before and after transplant were associated with elevated serum potassium, consistent with prior reports identifying renal dysfunction as a primary risk factor (6, 7). In our cohort, worsening renal function was associated with significant risk; on univariable analysis, patients with stage 3 post-operative AKI were more than two and a half times as likely to develop hyperkalemia compared to those without AKI. These findings are especially pertinent, given that AKI is the most common complication after LT, and severe AKI is associated with an increased risk of CKD in LT recipients (13, 14). Consistent with these observations, CKD stage at discharge was independently associated with hyperkalemia on multivariable analysis, with stages 3a, 3b, and 5 conferring significant risk. However, when a higher threshold for hyperkalemia was used (serum potassium > 5.5 mmol/L), only CKD stage 5 remained significantly associated, suggesting that advanced renal dysfunction may predict more severe potassium elevations.

While renal dysfunction was associated with hyperkalemia, we did not observe an independent association between CNI trough levels and hyperkalemia. However, this finding should not be interpreted as evidence that CNI exposure has limited clinical relevance to post-transplant hyperkalemia. CNIs are known to contribute to nephrotoxicity and hyperkalemia through afferent arteriole vasoconstriction and suppression of the renin-angiotensin-aldosterone system (15, 16). It is plausible that CNIs may have contributed to potassium elevations in our observed cohort, and several factors likely limited our ability to detect a dose-dependent relationship. Trough levels were assessed at post-operative day five and at discharge rather than at the time of the hyperkalemia event, and a single trough measurement may inadequately reflect cumulative CNI exposure or transient supratherapeutic levels. Moreover, CNI trough levels were routinely lowered in patients with renal dysfunction as part of standard post-transplant management and all patients received routine monitoring with dose adjustment, likely attenuating any detectable dose-response relationship in our cohort.

Even when adjusting for renal dysfunction, serum potassium prior to transplant and at the time of discharge were both predictive of hyperkalemia after discharge, despite group medians remaining within normal limits. While higher baseline serum potassium has been associated with intraoperative hyperkalemia, its relationship with post-transplant hyperkalemia has not been well characterized (4). Our data suggest that even modest elevations in serum potassium may reflect subclinical impairment in potassium handling. Under the physiological stress of LT and initiation of immunosuppression after transplant, patients with slight serum potassium elevations commonly progress to clinically significant hyperkalemia.

Importantly, several of the predictors identified in our analysis, including CKD stage and baseline serum potassium levels, are readily identifiable in routine clinical practice and available at or before the time of discharge. While hospital readmissions with hyperkalemia as the primary diagnosis were uncommon in our cohort (4%), hyperkalemia was a secondary issue in 20% of readmissions and was observed in nearly half of our cohort. These findings are especially important when considering the nationwide push to minimize the length of stay after liver transplant, since the peak incidence of hyperkalemia occurs after discharge for most patients. Outpatient management of hyperkalemia is further complicated by cost, prior authorization requirements, and limited availability of potassium-lowering medications at outpatient pharmacies, all of which delay treatment when it is most needed. Therefore, systems and targeted interventions need to be established to safeguard patients after discharge. Patients with advanced CKD, elevated pre-transplant or discharge potassium, or diabetes mellitus could be flagged for intensified serum potassium monitoring shortly after discharge. Because the cumulative incidence of hyperkalemia rose most rapidly within the first two weeks after discharge, surveillance efforts may be most impactful during this period. These efforts may include dietary counseling and consideration of prophylactic potassium-binding therapy and other medications as part of the discharge plan (17–19).

This study has several strengths. Our cohort is the largest to date examining hyperkalemia after discharge in LT recipients and provides a robust sensitivity analysis across alternative hyperkalemia thresholds. Importantly, we provide detailed insight into the timing of hyperkalemia onset, which has not been previously reported. However, several limitations must be considered. First, given the study’s single-center setting, our findings may not be generalizable to centers with different protocols. SLK recipients also represented a small subgroup (n=54) that was underpowered for separate analysis. Although previous data demonstrate improved renal function among SLK recipients compared to isolated liver transplant recipients, particularly among those with pre-transplant renal dysfunction (20, 21), SLK status was predictive of hyperkalemia in our cohort. Given these findings, future research should be directed towards SLK recipients. Factors such as medication adherence also could not be measured, and hyperkalemia-related readmissions occurring at outside hospitals may have been incompletely captured, likely resulting in underestimation of its true incidence and readmission rate. Post-transplant dietary intake was also not measured. Because dietary potassium restriction is routinely indicated following transplantation and a cornerstone of hyperkalemia management, variations in diet could have affected the observed incidence of hyperkalemia (22, 23). Confounding by indication also cannot be fully excluded for potassium-binding agents, which were likely initiated in response to rising or borderline potassium levels, or for CNI trough levels, which were lower in patients with renal dysfunction and adjusted in response to fluctuating renal function. Finally, universal exposure to trimethoprim-sulfamethoxazole precluded assessment of its contribution to hyperkalemia, despite its known association with potassium elevation in transplant recipients (24).

In conclusion, hyperkalemia is very common within the first three months following LT discharge, with most events occurring in the first two weeks of outpatient follow-up. Patients with higher baseline serum potassium levels or underlying renal dysfunction appear to be at the greatest risk. These findings support the development of risk-stratified surveillance and management guidelines for patients after liver transplant to reduce hyperkalemia-related morbidity and healthcare utilization.

## Data Availability

The raw data supporting the conclusions of this article will be made available by the authors, without undue reservation.
