# Supplementary material for: Out of sight, out of mind, but at high risk: incidence, timing and risk factors for hyperkalemia after liver transplant discharge
Source: Front Immunol. 2026 Oct 7;17:1924687. doi: 10.3389/fimmu.2026.1924687 (PMC13682998; doi:10.3389/fimmu.2026.1924687)
Supplement: Supplementary file 1 [file SupplementaryFile1.docx]

Supplementary Material

**Supplementary Figure S1.** Study population workflow.

**Supplementary Table S1.** Baseline demographics and transplant clinical characteristics: sensitivity analysis (serum potassium > 5.5 mmol/L).

**Supplementary Table S2.** Perioperative characteristics, medications, and laboratory values: sensitivity analysis (serum potassium > 5.5 mmol/L).

**Supplementary Table S3.** Univariable and multivariable Cox regression: sensitivity analysis (serum potassium > 5.5 mmol/L).

**Supplementary Table S4.** Baseline demographics and transplant clinical characteristics: isolated liver transplant recipients.

**Supplementary Table S5.** Perioperative characteristics, medications, and laboratory values: isolated liver transplant recipients.

**Supplementary Table S6.** Univariable and multivariable Cox regression: isolated liver transplant recipients.

**Supplementary Figure S1.** Study population workflow.

**Supplementary Table S1.** Baseline demographics and transplant clinical characteristics, stratified by hyperkalemia status (serum potassium > 5.5 mmol/L).

|  | **Hyperkalemia**  **n=153** | **No Hyperkalemia**  **n=391** | **p-value** |
| --- | --- | --- | --- |
| **Age at transplant** (years) | 59 (51-65) | 58 (49-65) | 0.58 |
| **Sex** |  |  | 0.13 |
| **Female** | 53 (35%) | 163 (42%) |  |
| **Male** | 100 (65%) | 228 (58%) |  |
| **Race/Ethnicity** |  |  | 0.14 |
| **White** | 50 (33%) | 169 (43%) |  |
| **Black** | 8 (5%) | 17 (4%) |  |
| **Hispanic/Latino** | 68 (44%) | 140 (36%) |  |
| **Asian** | 24 (16%) | 54 (14%) |  |
| **American Indian/**  **Alaska Native** | 3 (2%) | 5 (1%) |  |
| **Native Hawaiian/**  **Other Pacific Islander** | 0 (0%) | 6 (2%) |  |
| **BMI** | 27.8 (24.1-32.3) | 27.9 (24.1-32.7) | 0.95 |
| **Comorbidities** |  |  |  |
| **Diabetes mellitus** | 54 (35%) | 80 (20%) | <0.001 |
| **Hypertension** | 42 (27%) | 88 (23%) | 0.22 |
| **Hyperlipidemia** | 18 (12%) | 50 (13%) | 0.75 |
| **Etiology** |  |  | 0.39 |
| **Alcohol-associated** | 71 (46%) | 147 (38%) |  |
| **HBV** | 7 (5%) | 29 (7%) |  |
| **HCV** | 18 (12%) | 39 (10%) |  |
| **Cholestasis** | 9 (6%) | 35 (9%) |  |
| **Autoimmune** | 1 (1%) | 8 (2%) |  |
| **MASLD/MASH** | 34 (22%) | 88 (23%) |  |
| **Malignancy** | 2 (1%) | 8 (2%) |  |
| **Other** | 11 (7%) | 37 (9%) |  |
| **Transplant Details** |  |  |  |
| **Laboratory MELD at**  **transplant** | 29 (19-36) | 24 (13-33) | <0.001 |
| **SLK** | 20 (13%) | 34 (9%) | 0.13 |
| **Donor Age** (years) | 40 (30-48) | 41 (30-53) | 0.30 |
| **Donor Type** |  |  | 0.52 |
| **DBD** | 107 (70%) | 274 (70%) |  |
| **DCD** | 27 (18%) | 57 (15%) |  |
| **Living** | 19 (12%) | 60 (15%) |  |
| **NMP** | 42 (27%) | 111 (28%) | 0.83 |
| **CIT** (hours) | 8.4 (6.3-11.5) | 7.8 (5.7-11.5) | 0.17 |
| **EBL** (L) | 3.0 (1.5-5.4) | 2.0 (1.0-4.5) | <0.001 |

*Abbreviations: BMI (Body mass index), HBV (Hepatitis B virus), HCV (Hepatitis C virus), MASLD/MASH (Metabolic dysfunction-associated steatotic liver disease/Metabolic dysfunction-associated steatohepatitis), MELD (Model for End-Stage Liver Disease), SLK (Simultaneous liver-kidney transplant), DBD (Donation after brain death), DCD (Donation after circulatory death), NMP (Normothermic machine perfusion), CIT (Cold ischemia time), EBL (Estimated blood loss)*

**Supplementary Table S2.** Perioperative characteristics, medications, and laboratory values, stratified by hyperkalemia status (serum potassium > 5.5 mmol/L).

|  | Hyperkalemia  n=153 | No Hyperkalemia  n=391 | p-value |
| --- | --- | --- | --- |
| Dialysis |  |  |  |
| Dialysis prior to transplant | 45 (29%) | 70 (18%) | 0.003 |
| Required inpatient dialysis  after transplant | 58 (38%) | 108 (28%) | 0.02 |
| Dialysis after discharge | 25 (16%) | 29 (7%) | 0.002 |
| Post-Operative AKI* |  |  |  |
| Any | 137 (90%) | 295 (75%) | <0.001 |
| Stage 1† | 38 (28%) | 100 (34%) |  |
| Stage 2† | 24 (18%) | 55 (19%) |  |
| Stage 3† | 75 (55%) | 140 (47%) |  |
| EAD | 37 (24%) | 66 (17%) | 0.05 |
| LOS | 13 (8-22) | 10 (7-18) | 0.01 |
| Discharge Diet |  |  | 0.07 |
| Regular | 86 (56%) | 255 (65%) |  |
| Renal | 32 (21%) | 52 (13%) |  |
| Diabetic | 20 (13%) | 57 (15%) |  |
| Other | 15 (10%) | 27 (7%) |  |
| Medications Prior to Transplant |  |  |  |
| Bicarbonate | 29 (19%) | 45 (12%) | 0.02 |
| Patiromer calcium sorbitex | 1 (1%) | 1 (0.3%) | 0.49 |
| Sodium polystyrene sulfonate | 2 (1%) | 0 (0%) | 0.02 |
| Sodium zirconium  cyclosilicate | 18 (12%) | 28 (7%) | 0.08 |
| Furosemide During Transplant | 106 (69%) | 264 (68%) | 0.69 |
| Medications Prior to Discharge |  |  |  |
| Bicarbonate | 36 (24%) | 56 (14%) | 0.01 |
| Patiromer calcium sorbitex | 0 (0%) | 1 (0.3%) | 0.53 |
| Sodium polystyrene sulfonate | 1 (1%) | 3 (1%) | 0.89 |
| Sodium zirconium  cyclosilicate | 45 (29%) | 83 (21%) | 0.04 |
| Medications at Discharge |  |  |  |
| ACE inhibitor | 1 (1%) | 2 (1%) | 0.84 |
| Beta blocker | 24 (16%) | 70 (18%) | 0.54 |
| Furosemide | 27 (18%) | 74 (19%) | 0.73 |
| Insulin | 61 (40%) | 114 (29%) | 0.02 |
| Spironolactone | 0 (0%) | 2 (1%) | 0.38 |
| Trimethoprim-  sulfamethoxazole | 139 (91%) | 347 (89%) | 0.48 |
| Medications After Discharge |  |  |  |
| Bicarbonate | 33 (22%) | 48 (12%) | 0.006 |
| Sodium polystyrene  sulfonate | 2 (1%) | 4 (1%) | 0.78 |
| Sodium zirconium  cyclosilicate | 14 (9%) | 19 (5%) | 0.06 |
| One Month Pre-Transplant Labs | | | |
| Creatinine‡ | 1.1 (0.8-1.5) | 0.9 (0.7-1.2) | <0.001 |
| eGFR‡ | 66 (49-98) | 83 (59-103) | <0.001 |
| Potassium | 4.4 (4.0-4.9) | 4.0 (3.8-4.4) | <0.001 |
| One Week Pre-Transplant Labs |  |  |  |
| Creatinine‡ | 1.2 (0.8-1.7) | 0.9 (0.8-1.3) | 0.001 |
| eGFR‡ | 52 (30-90) | 77 (46-100) | <0.001 |
| Potassium | 4.2 (3.8-4.6) | 3.9 (3.6-4.2) | <0.001 |
| Tacrolimus Trough POD5 | 5.9 (4.2-7.8) | 6.2 (4.6-8.2) | 0.12 |
| Labs at Discharge |  |  |  |
| Tacrolimus trough | 7.5 (5.9-9.3) | 7.6 (6.0-9.3) | 0.77 |
| eGFR§ | 77 (54-99) | 89 (63-104) | 0.005 |
| AST | 26 (18-38) | 31 (20-51) | 0.004 |
| ALT | 76 (34-128) | 96 (40-171) | 0.02 |
| Potassium | 4.2 (3.9-4.6) | 4.1 (3.8-4.5) | 0.03 |
| CKD Stage at Discharge∥ |  |  | <0.001 |
| Stage 1 | 38 (25%) | 155 (40%) |  |
| Stage 2 | 38 (25%) | 98 (25%) |  |
| Stage 3a | 26 (17%) | 58 (15%) |  |
| Stage 3b | 17 (11%) | 29 (7%) |  |
| Stage 4 | 8 (5%) | 18 (5%) |  |
| Stage 5 | 26 (17%) | 33 (8%) |  |

*Patients with pre-transplant dialysis were considered AKI stage 3

†Of patients with post-operative AKI

‡Patients on dialysis prior to transplant were excluded

§Patients on inpatient dialysis after transplant were excluded

∥Patients on dialysis at or following discharge were considered CKD stage 5

Note: Among patients with hyperkalemia, post-discharge medications are those initiated prior to hyperkalemia onset.

*Abbreviations: AKI (Acute kidney injury), EAD (Early allograft dysfunction), LOS (length of stay), ACE (Angiotensin-converting enzyme), eGFR (estimated Glomerular Filtration Rate), POD (Post-operative day), AST (Aspartate aminotransferase), ALT (Alanine aminotransferase), CKD (Chronic kidney disease)*

**Supplementary Table S3.** Univariable and multivariable Cox regression for time to first hyperkalemia event (serum potassium > 5.5 mmol/L) within three-months of post-transplant discharge.

|  | Univariable  HR (95% CI) | p-value | Multivariable  aHR (95% CI) | p-value |
| --- | --- | --- | --- | --- |
| Patient Characteristics |  |  |  |  |
| Age at transplant  (per year increase) | 1.01 (1.00-1.02) | 0.20 |  |  |
| Sex, female reference | 1.29 (0.93-1.81) | 0.13 |  |  |
| Diabetes mellitus | 1.86 (1.33-2.59) | <0.001 | 1.87 (1.33-2.64) | <0.001 |
| Liver Disease Characteristics | | | | |
| Laboratory MELD at transplant  (per point increase) | 1.03 (1.01-1.04) | 0.001 | 1.02 (1.00-1.03) | 0.06 |
| Potassium  One week pre-transplant | 1.85 (1.48-2.31) | <0.001 | 1.65 (1.28-2.13) | <0.001 |
| Transplant Characteristics | | | | |
| EBL  (per liter increase) | 1.04 (1.01-1.07) | 0.01 | 1.02 (0.99-1.05) | 0.13 |
| Renal Function |  |  |  |  |
| CKD Stage at Discharge, Stage 1 reference* | | | | |
| Stage 2 | 1.51 (0.96-2.37) | 0.07 | 1.20 (0.75-1.92) | 0.45 |
| Stage 3a | 1.70 (1.03-2.80) | 0.04 | 1.26 (0.75-2.12) | 0.37 |
| Stage 3b | 2.11 (1.19-3.75) | 0.01 | 1.71 (0.94-3.11) | 0.08 |
| Stage 4 | 1.89 (0.88-4.04) | 0.10 | 1.50 (0.69-3.24) | 0.30 |
| Stage 5 | 2.36 (1.42-3.92) | 0.001 | 1.91 (1.13-3.24) | 0.02 |
| Laboratory Values at Discharge | | | | |
| Potassium  (per one unit increase) | 1.56 (1.10-2.20) | 0.01 | 1.42 (0.99-2.03) | 0.06 |

*Patients on dialysis at or following discharge were considered CKD stage 5

*Abbreviations: MELD (Model for End-Stage Liver Disease), EBL (Estimated blood loss), CKD (Chronic kidney disease)*

**Supplementary Table S4.** Baseline demographics and transplant clinical characteristics, stratified by hyperkalemia status (serum potassium > 5.0 mmol/L) among isolated liver transplant recipients.

|  | **Hyperkalemia**  **n=234** | **No Hyperkalemia**  **n=256** | **p-value** |
| --- | --- | --- | --- |
| **Age at transplant** (years) | 60 (51-65) | 58 (49-65) | 0.08 |
| **Sex** |  |  | 0.09 |
| **Female** | 85 (36%) | 112 (44%) |  |
| **Male** | 149 (64%) | 144 (56%) |  |
| **Race/Ethnicity** |  |  | 0.20 |
| **White** | 80 (34%) | 117 (46%) |  |
| **Black** | 9 (4%) | 12 (5%) |  |
| **Hispanic/Latino** | 95 (41%) | 92 (36%) |  |
| **Asian** | 41 (18%) | 30 (12%) |  |
| **American Indian/**  **Alaska Native** | 7 (3%) | 1 (0.4%) |  |
| **Native Hawaiian/**  **Other Pacific Islander** | 2 (1%) | 4 (2%) |  |
| **BMI** | 27.9 (23.8-32.3) | 28.0 (24.6-32.8) | 0.55 |
| **Comorbidities** |  |  |  |
| **Diabetes mellitus** | 69 (29%) | 49 (19%) | <0.001 |
| **Hypertension** | 56 (24%) | 56 (22%) | 0.59 |
| **Hyperlipidemia** | 31 (13%) | 28 (11%) | 0.43 |
| **Etiology** |  |  | 0.045 |
| **Alcohol-associated** | 98 (42%) | 96 (38%) |  |
| **HBV** | 12 (5%) | 23 (9%) |  |
| **HCV** | 31 (13%) | 24 (9%) |  |
| **Cholestasis** | 12 (5%) | 28 (11%) |  |
| **Autoimmune** | 2 (1%) | 7 (3%) |  |
| **MASLD/MASH** | 56 (24%) | 53 (21%) |  |
| **Malignancy** | 3 (1%) | 7 (3%) |  |
| **Other** | 20 (9%) | 18 (7%) |  |
| **Transplant Details** |  |  |  |
| **Laboratory MELD at**  **transplant** | 27 (15-35) | 23 (13-32) | 0.03 |
| **Donor Age** (years) | 42 (31-51) | 40 (30-52) | 0.70 |
| **Donor Type** |  |  | 0.31 |
| **DBD** | 167 (71%) | 168 (66%) |  |
| **DCD** | 35 (15%) | 41 (16%) |  |
| **Living** | 32 (14%) | 47 (18%) |  |
| **NMP** | 71 (30%) | 80 (31%) | 0.83 |
| **CIT** (hours) | 7.8 (6.0-11.4) | 7.5 (5.0-11.2) | 0.23 |
| **EBL** (L) | 2.5 (1.5-4.7) | 2.0 (1.0-4.3) | 0.02 |

*Abbreviations: BMI (Body mass index), HBV (Hepatitis B virus), HCV (Hepatitis C virus), MASLD/MASH (Metabolic dysfunction-associated steatotic liver disease/Metabolic dysfunction-associated steatohepatitis), MELD (Model for End-Stage Liver Disease), DBD (Donation after brain death), DCD (Donation after circulatory death), NMP (Normothermic machine perfusion), CIT (Cold ischemia time), EBL (Estimated blood loss)*

**Supplementary Table S5.** Perioperative characteristics, medications, and laboratory values, stratified by hyperkalemia status (serum potassium > 5.0 mmol/L) among isolated liver transplant recipients.

|  | Hyperkalemia  n=234 | No Hyperkalemia  n=256 | p-value |
| --- | --- | --- | --- |
| Dialysis |  |  |  |
| Dialysis prior to transplant | 48 (21%) | 27 (11%) | 0.002 |
| Required inpatient dialysis  after transplant | 86 (37%) | 50 (20%) | <0.001 |
| Dialysis after discharge | 30 (13%) | 16 (6%) | 0.01 |
| Post-Operative AKI* |  |  |  |
| Any | 204 (87%) | 181 (71%) | <0.001 |
| Stage 1† | 67 (33%) | 71 (39%) |  |
| Stage 2† | 34 (17%) | 44 (24%) |  |
| Stage 3† | 103 (50%) | 66 (36%) |  |
| EAD | 54 (23%) | 39 (15%) | 0.03 |
| LOS | 12 (8-20) | 10 (7-16) | <0.001 |
| Discharge Diet |  |  | 0.26 |
| Regular | 144 (62%) | 173 (68%) |  |
| Renal | 40 (17%) | 28 (11%) |  |
| Diabetic | 32 (14%) | 36 (14%) |  |
| Other | 18 (8%) | 19 (7%) |  |
| Medications Prior to Transplant |  |  |  |
| Bicarbonate | 31 (13%) | 21 (8%) | 0.07 |
| Patiromer calcium sorbitex | 2 (1%) | 0 (0%) | 0.14 |
| Sodium polystyrene sulfonate | 2 (1%) | 0 (0%) | 0.14 |
| Sodium zirconium  cyclosilicate | 24 (10%) | 16 (6%) | 0.11 |
| Furosemide During Transplant | 168 (72%) | 166 (65%) | 0.10 |
| Medications Prior to Discharge |  |  |  |
| Bicarbonate | 44 (19%) | 29 (11%) | 0.02 |
| Patiromer calcium sorbitex | 0 (0%) | 1 (0.4%) | 0.34 |
| Sodium polystyrene sulfonate | 1 (0.4%) | 1 (0.4%) | 0.95 |
| Sodium zirconium  cyclosilicate | 62 (27%) | 43 (17%) | 0.01 |
| Medications at Discharge |  |  |  |
| ACE inhibitor | 2 (1%) | 1 (0.4%) | 0.51 |
| Beta blocker | 50 (21%) | 39 (15%) | 0.08 |
| Furosemide | 39 (17%) | 52 (20%) | 0.30 |
| Insulin | 79 (34%) | 77 (30%) | 0.38 |
| Spironolactone | 1 (0.4%) | 1 (0.4%) | 0.95 |
| Trimethoprim-  sulfamethoxazole | 207 (88%) | 227 (89%) | 0.94 |
| Medications After Discharge |  |  |  |
| Bicarbonate | 60 (26%) | 28 (11%) | <0.001 |
| Sodium polystyrene  sulfonate | 4 (2%) | 2 (1%) | 0.35 |
| Sodium zirconium  cyclosilicate | 17 (7%) | 10 (4%) | 0.10 |
| One Month Pre-Transplant Labs | | | |
| Creatinine‡ | 1.01 (0.80-1.36) | 0.87 (0.69-1.10) | 0.001 |
| eGFR‡ | 71 (52-98) | 88 (61-112) | <0.001 |
| Potassium | 4.3 (3.9-4.7) | 3.9 (3.7-4.3) | <0.001 |
| One Week Pre-Transplant Labs |  |  |  |
| Creatinine‡ | 1.04 (0.79-1.46) | 0.87 (0.72-1.21) | <0.001 |
| eGFR‡ | 78 (49-98) | 89 (64-105) | 0.002 |
| Potassium | 4.1 (3.8-4.4) | 3.9 (3.6-4.2) | <0.001 |
| Tacrolimus Trough POD5 | 5.8 (4.1-8.0) | 6.3 (4.7-8.1) | 0.05 |
| Labs at Discharge |  |  |  |
| Tacrolimus trough | 7.6 (5.9-9.2) | 7.7 (6.0-9.5) | 0.60 |
| eGFR§ | 78 (56-99) | 93 (66-107) | <0.001 |
| AST | 27 (18-43) | 34 (21-54) | 0.002 |
| ALT | 71 (32-139) | 97 (53-179) | <0.001 |
| Potassium | 4.2 (3.9-4.6) | 4.1 (3.8-4.4) | 0.001 |
| CKD Stage at Discharge∥ |  |  | <0.001 |
| Stage 1 | 65 (28%) | 114 (45%) |  |
| Stage 2 | 57 (24%) | 69 (27%) |  |
| Stage 3a | 45 (19%) | 33 (13%) |  |
| Stage 3b | 26 (11%) | 14 (5%) |  |
| Stage 4 | 9 (4%) | 9 (4%) |  |
| Stage 5 | 32 (14%) | 17 (7%) |  |

*Patients with pre-transplant dialysis were considered AKI stage 3

†Of patients with post-operative AKI

‡Patients on dialysis prior to transplant were excluded

§Patients on inpatient dialysis after transplant were excluded

∥Patients on dialysis at or following discharge were considered CKD stage 5

Note: Among patients with hyperkalemia, post-discharge medications are those initiated prior to hyperkalemia onset.

*Abbreviations: AKI (Acute kidney injury), EAD (Early allograft dysfunction), LOS (length of stay), ACE (Angiotensin-converting enzyme), eGFR (estimated Glomerular Filtration Rate), POD (Post-operative day), AST (Aspartate aminotransferase), ALT (Alanine aminotransferase), CKD (Chronic kidney disease)*

**Supplementary Table S6.** Univariable and multivariable Cox regression for time to first hyperkalemia event (serum potassium > 5.0 mmol/L) within three months of post-transplant discharge among isolated liver transplant recipients.

|  | Univariable  HR (95% CI) | p-value | Multivariable  aHR (95% CI) | p-value |
| --- | --- | --- | --- | --- |
| Patient Characteristics |  |  |  |  |
| Age at transplant  (per year increase) | 1.01 (1.00-1.02) | 0.03 | 1.01 (1.00-1.02) | 0.21 |
| Sex, female reference | 1.29 (0.99-1.68) | 0.06 | 1.32 (1.00-1.73) | 0.048 |
| Diabetes mellitus | 1.45 (1.09-1.92) | 0.01 | 1.40 (1.04-1.87) | 0.03 |
| Liver Disease Characteristics | | | | |
| Laboratory MELD at transplant  (per point increase) | 1.01 (1.00-1.02) | 0.03 | 1.01 (0.99-1.02) | 0.34 |
| Potassium  One week pre-transplant | 1.70 (1.41-2.04) | <0.001 | 1.56 (1.26-1.92) | <0.001 |
| Transplant Characteristics | | | | |
| EAD | 1.38 (1.02-1.87) | 0.04 | 1.15 (0.83-1.59) | 0.40 |
| Renal Function |  |  |  |  |
| CKD Stage at Discharge, Stage 1 reference* | | | | |
| Stage 2 | 1.35 (0.94-1.92) | 0.10 | 1.04 (0.71-1.53) | 0.84 |
| Stage 3a | 1.89 (1.29-2.76) | 0.001 | 1.56 (1.04-2.34) | 0.03 |
| Stage 3b | 2.47 (1.57-3.90) | <0.001 | 2.21 (1.36-3.61) | 0.001 |
| Stage 4 | 1.65 (0.82-3.31) | 0.16 | 1.20 (0.58-2.47) | 0.62 |
| Stage 5 | 2.02 (1.32-3.08) | 0.001 | 1.75 (1.10-2.77) | 0.02 |
| Laboratory Values at Discharge | | | | |
| Potassium  (per one unit increase) | 1.76 (1.32-2.34) | <0.001 | 1.59 (1.18-2.15) | 0.002 |

*Patients on dialysis at or following discharge were considered CKD stage 5

*Abbreviations: MELD (Model for End-Stage Liver Disease), EAD (Early allograft dysfunction), CKD (Chronic kidney disease)*
